# First report of *VGSC* mutations for resistance to synthetic pyrethroids in brown dog ticks (*Rhipicephalus sanguineus* sensu stricto) from Brazil

**DOI:** 10.1186/s13071-025-07099-x

**Published:** 2025-11-21

**Authors:** Hayden Kleinschmidt, Nathan E. Stone, Rebecca Ballard, Natalie B. Thornton, Grant L. Pemberton, Simone Becker, Guilherme M. Klafke, David M. Wagner, Joseph D. Busch

**Affiliations:** 1 Pathogen and Microbiome Institute, Northern Arizona University, 1395 S. Knoles Dr. Bldg 56, Flagstaff, AZ 86011-4073 USA; 2 Instituto de Pesquisas Veterinárias Desidério Finamor, Estrada do Conde, 6000, Eldorado do Sul, 92990-000 Brazil

**Keywords:** Resistance, Voltage-gated sodium channel, Synthetic pyrethroids, Deltamethrin, Sequencing, Rocky Mountain spotted fever, Brown dog tick

## Abstract

**Background:**

Brown dog ticks, *Rhipicephalus sanguineus* sensu lato (s.l.), have spread globally and are an important vector of multiple pathogens affecting both dogs and humans. The control of these ticks on dogs and human dwellings often relies on synthetic pyrethroids, a chemical class of acaricides that targets the voltage-gated sodium channel (VGSC) protein of arthropod nerve cells, causing influxes of sodium and ultimately, paralysis. Invasive *Rhipicephalus sanguineus* s.l. ticks in the Americas can be broadly grouped into two main lineages: temperate and tropical (*Rh. sanguineus* sensu stricto (s.s.) and *Rh. linnaei*, respectively). Phenotypic resistance to synthetic pyrethroids in a verified *Rh. sanguineus* s.s. population has only been reported in the state of Rio Grande do Sul, Brazil. The objectives of our study were to 1) screen a small number (*n* = 10) of *Rh. sanguineus* s.s. from Brazil to check for *VGSC* gene mutations known to be associated with resistance to synthetic pyrethroids in ticks and other arthropods, and 2) provide molecular confirmation that all ticks were *Rh. sanguineus* s.s.

**Methods:**

We used next-generation DNA sequencing methods to analyze the *VGSC* gene and mitochondrial loci (*12S*, *16S*, *COI*) of 10 brown dog ticks sampled from a stray dog in the Restinga sub-district of Porto Alegre, Rio Grande do Sul, Brazil. The progeny of other ticks from this animal had previously been shown to display a low level of resistance to deltamethrin.

**Results:**

Analysis of mitochondrial genes confirmed these ticks were *Rh. sanguineus* s.s. We identified two known resistance mutations in domain II segments 4 and 5 of the *VGSC* gene (C190A and G215T). These specific mutations have not been reported previously in any brown dog tick lineages from the Americas, and this is the first case of *VGSC* mutations described from ticks confirmed to be *Rh. sanguineus* s.s. using genetic analyses.

**Conclusions:**

The discovery of these mutations in *Rh. sanguineus* s.s. is important for the effective management of ticks on dogs in Brazil and other countries where brown dog tick infestations occur.

**Graphical Abstract:**

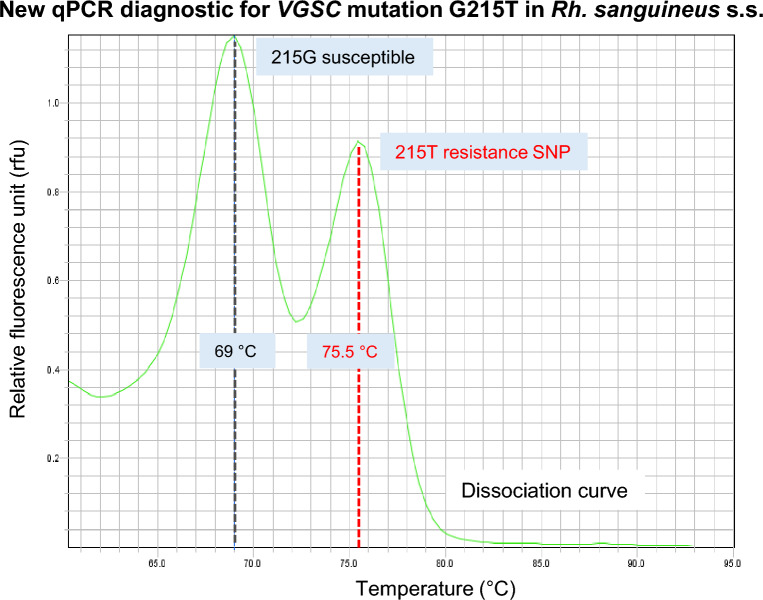

**Supplementary Information:**

The online version contains supplementary material available at 10.1186/s13071-025-07099-x.

## Background

Brown dog ticks, *Rhipicephalus sanguineus* sensu lato (s.l.), comprise a group of related tick species [1, 2] that have spread globally with domestic dogs via human transport. These ticks are important vectors of the canine pathogens *Babesia vogeli* and *Ehrlichia canis* [3] and transmit a number of rickettsial species in the spotted fever group to humans, including *Rickettsia rickettsii* (causative agent of Rocky Mountain spotted fever) [4, 5], *R. massiliae* in the southwestern USA and Mexico [6], and *R. conorii* in Europe [7]. Current methods for disease prevention rely heavily on chemical control of ticks on dogs. Brown dog ticks can live in and around human dwellings and prefer to feed on domestic dogs throughout their three life stages [8–10]. Two main lineages have spread throughout the Americas: *Rh. sanguineus* sensu stricto (s.s.), commonly referred to as “temperate” lineage ticks [11], and the “tropical” lineage, recently named *Rh. linnaei* in some parts of the world [12]. Other *Rh. sanguineus* s.l. taxa are found in Asia and Europe [1, 13]. In Brazil, *Rh. sanguineus* s.l. has been shown to carry *R. rickettsii* [14] and is a suspected vector for transmission to humans [15]. Stray dogs in urban environments such as Porto Alegre facilitate the persistence and spread of *Rh. sanguineus* s.s., which is the most common tick species found on dogs in the state of Rio Grande do Sul [16].

Synthetic pyrethroids are a chemical class of acaricides commonly used throughout the world to control tick infestations [17], including brown dog ticks [18]. Resistance to synthetic pyrethroids such as deltamethrin, permethrin, and cypermethrin is usually due to nonsynonymous single-nucleotide polymorphisms (SNPs) in the voltage-gated sodium channel (*VGSC*) gene that is essential for proper nerve functioning in arthropod species [19], although metabolic detoxification can also play a role [20–22]. Phenotypic resistance to synthetic pyrethroids has been described in various *Rh. sanguineus* s.l. tick populations throughout the Americas, including Brazil [16], Panama [22], the Caribbean [23], and the USA [18, 24]. Furthermore, a resistance SNP (T2134C) in domain III segment 6 of the *VGSC* gene has been described in *Rh. linnaei* samples from multiple states of the USA [25, 26], and this same mutation has been described in a synthetic pyrethroid-resistant population of *Rh. sanguineus* s.l. from Florida [27]. Additional SNPs conferring resistance to bioassays for deltamethrin and flumethrin have been reported in brown dog ticks from India, including C190A and G215T in domain II segments 4–5 of the *VGSC* [28]. The C190A and T2134A SNPs are common in a related tick, *Rh.* (*Boophilus*) *microplus*, due to frequent use of these chemicals to treat ticks on cattle [26, 29, 30]. However, little is known about mutations conferring acaricide resistance in *Rh. sanguineus* s.s. populations in Brazil despite the past use of synthetic pyrethroids there. In this study, we screened a small collection of brown dog ticks from Brazil [16] for any of the known resistance mutations in the *VGSC* gene.

## Methods

DNA was extracted from 10 brown dog ticks (three adult females and seven nymphs) leftover from a previous study [16] conducted by the Instituto de Pesquisas Veterinárias Desidério Finamor (IPVDF), Eldorado do Sul, Brazil (Additional file 1). These ticks (hereafter referred to as Restinga ticks) were collected from a single stray dog in the Restinga sub-district of Porto Alegre, Brazil [31]. Although the 10 ticks were not tested for phenotypic resistance to deltamethrin, six engorged females sampled from the same dog produced an F1 larval generation that demonstrated a low level of resistance to deltamethrin (resistance ratio 5.67) in larval packet tests. The history of acaricide exposure in prior generations leading to this field tick population is unknown.

All molecular procedures followed the methods from two previous studies [25, 26]; primers specific to *VGSC* targets were designed in Stone et al. [26] and updated in Thomas et al. [32]. Amplicon sequencing (AmpSeq) of three PCR targets was performed on each sample to obtain partial sequences of *VGSC* [25]. Because resistance to fipronil was detected in other brown dog tick (BDT) samples from the metropolitan area of Porto Alegre [16], we included an additional PCR target for the GABA-gated chloride channel (*GABA-Cl*), which contains mutations associated with resistance to fipronil and dieldrin [33–36]. The Restinga ticks had previously been identified as *Rh. sanguineus* s.s. on the basis of morphology according to the Barros-Battesti guide [31] and their location in far southern Brazil [37]. To confirm the species identity and provide a phylogeographic context, we generated one amplicon each for three mitochondrial genes (*12S rRNA*, *16S rRNA*, and *COI*). The seven targets were amplified in two multiplex PCRs as outlined in Stone et al. [25] and pooled within each individual, then indexed and sequenced on an Illumina NextSeq 1000 instrument using a P1 600 cycle (2 × 300) Reagent Kit with PhiX control (Illumina, San Diego, CA, USA, part# 20100981), with the goal of obtaining hundreds to thousands of reads per amplicon for each individual. Bioinformatics methods for removing errors, SNP calling, and identifying heterozygotes versus homozygotes are described in our recent study of *Rh. sanguineus* [25]. Mitochondrial sequences for *COI* were aligned against publicly available sequences of *Rh. sanguineus* s.l. using BioEdit (v7.1.3) [38] and a maximum likelihood phylogeny was inferred with IQ-TREE (v2.2.0.3) [39] with the “-bb” bootstrapping option (*n* = 1000) and the integrated ModelFinder method [40]. Presumptive resistance SNPs were mapped onto major phylogenetic clades using Adobe Illustrator. To confirm the presence of *VGSC* mutations in domain II segments 4–5, real-time genotyping PCR assays were performed [26, 32], followed by Sanger sequencing of domain II segments 4–5, as previously described [26]. Sequences for *COI*, *12S*, and *16S* were also aligned against a small set of reference sequences [25] and assigned a haplotype (Additional file 1). Finally, a new real-time genotyping assay for G215T was developed for this study (Additional file 1) following previously described methods [26, 41].

## Results

**Fig. 1:**
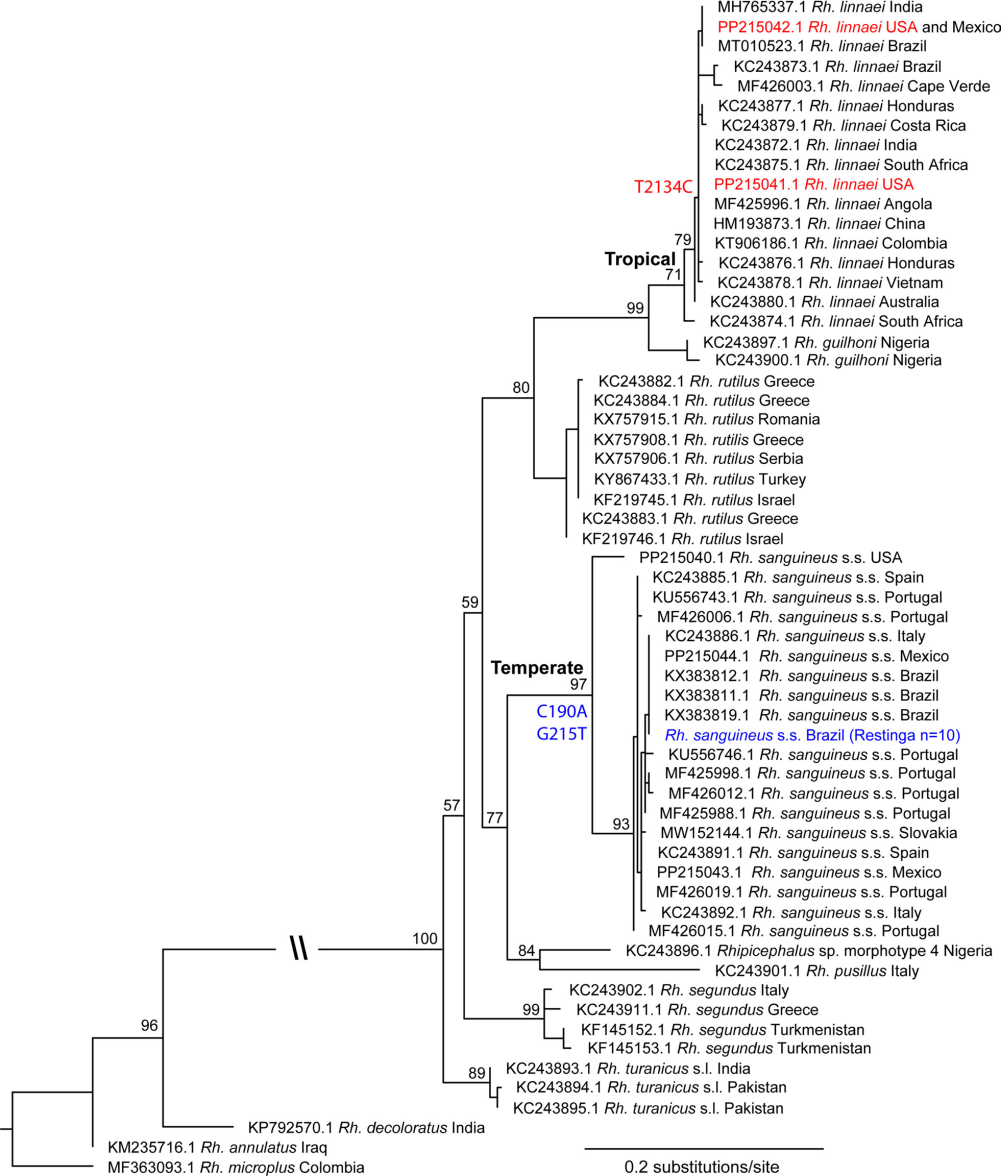
Maximum likelihood phylogenetic tree of *Rhipicephalus sanguineus* sensu lato based on mitochondrial *COI* partial sequence (346 bp), with resistance mutations mapped onto terminal branches. Two *Rh. sanguineus* s.l. clades that are invasive in South America (tropical and temperate) are labeled at interior nodes; bootstrap confidence values from 1000 permutations are labeled above major branches. Font colors denote ticks with resistance SNPs in *VGSC* domain II segments 4–5 (C190A or G215T; blue font) or domain III segment 6 (T2134C; red font)

Analysis of partial *COI* sequences (346 bp) grouped the Restinga ticks together in a clade with multiple *Rh. sanguineus* s.s. (temperate lineage) reference samples (Fig. 1), confirming the previous identification on the basis of morphology and geographical location. The sensu stricto classification was also confirmed by the *12S* (339 bp) and *16S* (215 bp) sequences (Additional file 1). All 10 Restinga ticks carried identical sequences at each of the three mitochondrial gene targets (Additional file 1). A total of 2 of the 10 ticks carried nonsynonymous SNPs in domain II segments 4–5 of the *VGSC*; both were heterozygotes (Additional file 1). The first (Rs_Restinga_04) carried the well-described synthetic pyrethroid resistance SNP C190A that leads to amino acid replacement L925I, and the second (Rs_Restinga_07) carried a putative resistance SNP G215T that leads to amino acid replacement G933V (aka G72V). The ticks included in this study were screened against an existing qPCR assay for the C190A SNP as well as the new G215T qPCR assay developed in this study, which confirmed the presence of these resistance genotypes. Both ticks carried only one of the resistance SNPs and were homozygous for susceptible states at other *VGSC* positions associated with resistance to synthetic pyrethroids in ticks (*i.e.,* nucleotide positions 170 and 2134). Sanger sequencing of all 10 ticks confirmed the qPCR results. Sequences from the *GABA-Cl* gene suggested the presence of three gene copies: *alpha*, *beta*, and *gamma* (Additional file 1), as reported previously for *Rh. sanguineus* s.l. [25]. Six Restinga ticks carried allele 2 of the *GABA-Cl-beta* copy (see Additional file 1; RsGABAchannel_beta_A02), which contains amino acid substitution A301S that is associated with dieldrin/fipronil resistance in other arthropods [42]. However, phenotypic resistance to fipronil was not tested in any Restinga samples, and it remains unknown if this allele is associated with resistance in *Rh. sanguineus* s.s.

## Discussion

The partial *COI* sequences verified that these Restinga tick samples were *Rh. sanguineus* s.s., which was expected because this species has been described previously in Rio Grande do Sul, Brazil [37], where the temperate climate is predicted to be favorable for this lineage [43]. Here, we report the first confirmed instance of *VGSC* resistance SNPs C190A and G215T from *Rh. sanguineus* s.s. ticks in the Americas. Both SNPs have been associated with synthetic pyrethroid resistance in other arthropods [19]; however, it remains unknown whether C190A and G215T convey resistance in *Rh. sanguineus* s.s. These mutations have been identified previously in two brown dog ticks from India [28], but mitochondrial lineage analysis was not reported in that study, so it is unknown if those ticks also were *Rh. sanguineus* s.s. Although the Restinga sample size is small, our findings underscore the urgent need to investigate *VGSC* SNPs in Brazil and elsewhere in South America by conducting in-depth studies to confirm phenotype–genotype correlations.

Synthetic pyrethroids have been widely used for tick (and flea) prevention on domestic dogs for > 25 years [18], and resistance SNPs in *Rh. linnaei* ticks sampled from companion animals are widespread in the USA [25]. The Restinga ticks were sampled from a stray dog with an unknown history of ectoparasite treatment; the presence of ticks carrying two independent resistance SNPs may indicate that prior tick generations had experienced synthetic pyrethroid exposure at some point, most likely when parasitizing pet dogs. It is also possible that metabolic detoxification [22] is an important mechanism of resistance in brown dog tick populations in Brazil. Regardless, the discovery of multiple resistance SNPs in *Rh. sanguineus* s.s. and *Rh. sanguineus* s.l. from different parts of the world emphasizes the need to develop alternative approaches for managing tick populations, such as anti-tick vaccines [44].

Mutations in the *VGSC* gene can have a strong phenotype–genotype correlation [30] that allows the prediction of synthetic pyrethroid resistance in field tick populations using only genotypes. We were not able to directly test for a phenotype–genotype correlation because no DNA was available from the F1 larvae used in larval packet tests (LPTs) [16]. Despite our small sample size, we note that a low deltamethrin resistance ratio (5.67) in the Restinga dog ticks is consistent with finding only two heterozygous ticks that carried putative *VGSC* resistance SNPs. When acaricides are used frequently and without a systematic management plan, a single heterozygous mutation conferring resistance against a widely used acaricide such as deltamethrin could lead to large-scale spread of resistant ticks in urban areas.

## Conclusion

This study provides the first molecular evidence of putative *VGSC* resistance mutations in *Rh. sanguineus* s.s. from South America, highlighting the importance of using applied genetic tools that can 1) identify additional nonsynonymous SNPs in the *VGSC* (*e.g.,* AmpSeq) and 2) rapidly screen for known mutations using real-time PCR [26, 32]. To accommodate this need for the G215T SNP, we developed a new assay in this study (Additional file 1). Both *Rh. sanguineus* s.s. and *Rh. linnaei* have colonized Brazil [37] and other countries in South America [45], and there is a need to monitor for resistance to synthetic pyrethroid acaricides used in the control of brown dog ticks. Future work in Brazil by IPVDF will expand the geographic scope of brown dog tick collections across both urban and rural areas, and a key objective is to integrate molecular analyses with standardized phenotypic assays (e.g., larval packet tests) to validate the predictive value of resistance-associated SNPs in the *VGSC*. These efforts will be complemented by epidemiological studies mapping the distribution of acaricide resistance and tick-borne pathogens. Together, these initiatives aim to establish an evidence-based framework for controlling *Rh. sanguineus* infestations in southern Brazil, particularly in densely populated urban areas. The use of molecular tools to monitor resistance levels (via genotype) will contribute to more effective management of tick populations, and by extension, reduce the occurrence of diseases transmitted by brown dog ticks.

## Supplementary Information

** Additional file 1: Tables S1, S2, and S3.**Genotypes generated for ten *Rhipicephalus sanguineus* sensu stricto ticks from Restinga, Brazil.

## Data Availability

The data supporting the conclusions of this article are included within the article and its additional file. All GenBank files are listed in Additional file 1.
